# Based on Histogram Analysis: ADC_aqp_ Derived from Ultra-high b-Value DWI could be a Non-invasive Specific Biomarker for Rectal Cancer Prognosis

**DOI:** 10.1038/s41598-020-67263-4

**Published:** 2020-06-23

**Authors:** Guangwen Zhang, Wanling Ma, Hui Dong, Jun Shu, Weihuan Hou, Yong Guo, Mian Wang, Xiaocheng Wei, Jialiang Ren, Jinsong Zhang

**Affiliations:** 1 Department of Radiology, Xijing Hospital, Fourth Military Medical University, Xi’an, Shaanxi P.R. China; 2 Research Equipment Management Center, General Hospital of Ningxia Medical University, Yinchuan, Ningxia, P.R. China; 3 Department of Gastrointestinal Pathology, Xijing Hospital, Fourth Military Medical University, Xi’an, Shaanxi P.R. China; 4 Department of Gastrointestinal Surgery, Xijing Hospital, Fourth Military Medical University, Xi’an, Shaanxi P.R. China; 5 MR Research China, GE Healthcare Greater China, Beijing, P.R. China

**Keywords:** Cancer imaging, Tumour biomarkers, Biomarkers, Oncology

## Abstract

Aquaporins (AQP) are not only water channel protein, but also potential prognostic indicator and therapeutic target for rectal cancer. Some previous studies have demonstrated the AQP expression could be estimated by ADC_aqp_ value derived from ultra-high b-value diffusion-weighted imaging (DWI). We aim to determine whether ADC_aqp_ could be a new and specific biomarker for indicating the AQP expression and prognostic factors of rectal cancer. 76 untreated patients with rectal cancer confirmed by colonoscopy biopsy were enrolled. ADC_aqp_ value was generated from ultra-high b-value DWI with five b-values (1700–3500 s/mm^2^). AQP (AQP1, 3 and 5)staining intensity was estimated by both of software (QuPath) and manual manner. The relationships between histogram features of ADC_aqp_ and AQP staining intensity were analyzed. The correlations between histogram features of ADC_aqp_ and differentiation degrees (good, moderate, poor), T stage (T1–2 vs T3–4), and lymph node status (N+ vs N−) were also evaluated respectively. The mean, 75^th^ percentile and 97.5^th^ percentile of ADC_aqp_ were correlated with AQP1 staining intensity (*r *= 0.237, 0.323 and 0.362, respectively, all *P *< 0.05) . No correlation was found between the histogram features of ADC_aqp_ and AQP3 or AQP5 staining intensity. The mean, 50^th^ percentile, 75^th^ percentile and 97.5^th^ percentile of ADC_aqp_ value exhibited significant differences between differentiation status (all *P* < 0.05). Histogram features of ADC_aqp_ value showed no significant differences in two subgroups of T stage and lymph node status (all *P* > 0.05). Histogram analysis showed that the ADC_aqp_ value derived from ultra-high b-value DWI of rectal cancer could reflect AQP1’s expression and rectal cancer’s malignancy degree. ADC_aqp_ might be a new imaging biomarker for evaluating rectal cancer.

## Introduction

Diffusion weighted imaging (DWI) is a technique which can depict water molecule movement *in vivo* depending on a pair of pulsed magnetic field gradients^1^. The diffusivity of water is quantitatively estimated by apparent diffusion coefficient (ADC) which is affected by the fast flow of water molecules in capillary vessels, slow movement in extracellular and intracellular compartments^2^ and slower passage through cell membranes by aquaporins (AQP)^3,4^. What excites researchers is that water transmembrane diffusivity can be estimated by using ultra-high b-value DWI and adequate effective diffusion time^5,6^.

Water transmembrane exchange is mainly mediated by AQP which is a highly conserved family of integral plasma membrane proteins^7^. Up to now, at least 13 AQPs been discovered and they are widely and diversely distributed and expressed at different organs and tumors in the role of water reabsorption^8^, brain-fluid homeostasis^9^, tumor cell migration^10^ and angiogenesis^11^, etc. Tomita *et al*.^12^ speculated that AQP1 may regulate the invasiveness of tumor cells by mediating the Wnt/β-catenin signal pathway and Yoshida *et al*.^13^ found that the overexpression of AQP1was an independently poor prognostic factor for stage II and III colon cancer. Byung *et al*.^14^ found the expressions of AQP1, 3 and 5 were related to the lymph node status in patients with colon cancer. *In vitro*, the pharmacological blockade of aquaporin-1 water channel by AqB013 could restrict migration and invasiveness of colon cancer cells and prevents endothelial tube formation^15^.These findings indicate that the AQP1 is not only a water channel protein, but also potential prognostic indicator and therapeutic target.

Mukherjee *et al*.^5^ demonstrated that AQP1’s overexpression could produce remarkable contrast in DWI both *in vitro* and immunodeficient mice with Chinese hamster ovary (CHO) cell xenografts. The correlation between AQP expression and ADC value acquired with ultra-high b-value DWI has been confirmed in patients with cerebral astrocytoma^16^ and rat models with diabetic nephropathy^17^. Histogram analysis which has been extensively applied into exploring medical imaging was demonstrated as a more accurate and informative method in assessment of tumorous characters than the mean value^18–21^.

Those aforementioned studies shed light on the possibility of using ultra-high b-value DWI technique with combining histogram analysis to investigate rectal cancer. Therefore, this study aims to investigate whether the histogram features of ultra-high b-value DWI of rectal cancer are correlated with AQP expression and prognostic related indicators (differentiation degree and TN stage).

## Results

### Inter-observer Reproducibility

All quantitative parameters involved in this study were acquired by two experts independently. Given the same estimation method applied for staining scoring of AQP1, 3 and 5, we only tested the ICC for the AQP1 but not for AQP3 and AQP5. The ICC analysis of the mean, 2.5^th^ percentile,25^th^ percentile,50^th^ percentile,75^th^ percentile,97.5^th^percentile and skewness of AQP1 staining score by QuPath showed good reliability (Table 1), while the kurtosis performed poorly. The AQP1 staining score acquired manually proved to be reliable (ICC = 0.807), but the scoring method with QuPath performed better than the manual method for most histogram features (Table 1). High reliability could be observed for every histogram feature of ADC_aqp_ value (all ICC > 0.930).

**Table 1:** The inter-observer reproducibility of all quantitative parameters between observer 1 and observer 2.

Quantitative parameter		ICC	Quantitative parameter		ICC
**AQP1 Score(QuPath)**	Mean	0.980	ADC_aqp_	Mean	0.993
	2.5^th^Per	0.957		2.5^th^Per	0.972
	25^th^Per	0.947		25^th^Per	0.983
	50^th^per	0.975		50^th^per	0.995
	75^th^ per	0.986		75^th^per	0.993
	97.5^th^per	0.985		97.5^th^per	0.993
	Kurtosis	0.422		Kurtosis	0.933
	Skewness	0.758		Skewness	0.962
**AQP1 Score (manual)**		0.807			

Per is the abbreviation of Percentile. ICC ≥ 0.75 is considered as good reliability.

### ADC_aqp_ and AQP immunohistochemistry

**Figure 1:**
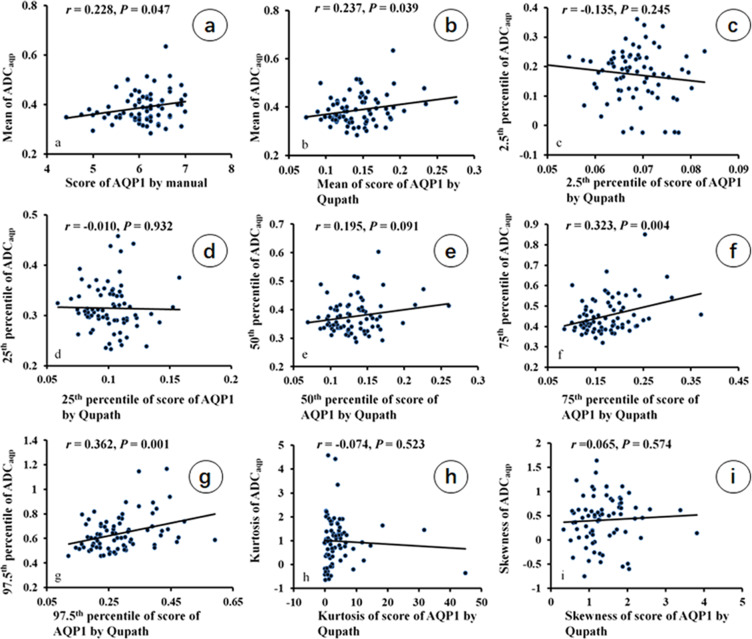
Correlation between histogram features of ADC_aqp_ and staining intensity of AQP1 by using manual analysis and QuPath (n = 76). The mean ADC_aqp_ value was correlated with the score of AQP1 IHC by manual analysis and the mean score of AQP1 IHC by QuPath (**a**,**b**). The75^th^ percentile and the 97.5^th^ percentile of ADC_aqp_ value was correlated with corresponding histogram features of AQP1 staining intensity by QuPath (**f**,**g**).

The correlation between ADC_aqp_ value and score of AQP1 staining intensity is shown in Fig. 1. The75^th^ percentile and the 97.5^th^ percentile of ADC_aqp_ value (0.452 ± 0.085 and 0.639 ± 0.142 μm^2^/ms) showed a significantly positive correlation with corresponding histogram features of AQP1 staining intensity by QuPath (75^th^ percentile of AQP1, 0.174 ± 0.052; 97.5^th^ percentile of AQP1, 0.285 ± 0.094)(*r* = 0.323 and 0.362, respectively, both *P* < 0.05; Fig. 1f,g). There were trend correlations between mean ADC_aqp_ value (0.388 ± 0.062μm^2^/ms) and manual AQP1 score (6.070 ± 0.543) (*r* = 0.228, *P* = 0.047; Fig. 1a), as well as the mean QuPath score (0.144 ± 0.036) (*r* = 0.237, *P* = 0.039; Fig. 1b). However, the 2.5^th^ percentile, the 25^th^ percentile, 50^th^ percentile, kurtosis and skewness of ADC_aqp_ value did not exhibit significant correlation with AQP1 staining intensity by QuPath (Fig. 1c,d,e,h,i). The mean AQP1 staining score with QuPath showed a significant correlation with the manual AQP1score (*r* = 0.685, *P* < 0.001, Supplementary Fig. S1). No histogram features of AQP3 or AQP5 staining intensity were found to be correlated with ADC_aqp_ value (Supplementary Table S1).

### ADC_aqp_ and rectal cancer differentiation

Of the 76 cases, 13 good, 53 moderate and 10 poor differentiated rectal cancers were confirmed by pathological examination. The mean, 50^th^ percentile, 75^th^ percentile and 97.5^th^ percentile of ADC_aqp_ value exhibited significant difference between the differentiation status of rectal cancer (all the *P* < 0.05, Table 2). In addition, these histogram features of ADC_aqp_ value tended to increase with the malignancy degree. However, it was only the 75^th^ percentile of ADC_aqp_ value which showed significant differences in post hoc multiple comparisons between three differentiation subgroups (*P* = 0.016, good vs moderate; *P* < 0.001, good vs poor; *P* = 0.002, moderate vs poor).And there was no significant difference for the 2.5^th^ percentile, 25^th^ percentile, kurtosis and skewness of ADC_aqp_ value between differentiation degrees(*P* > 0.05).

**Table 2:** Correlation between ADCaqp histogram features and histological differentiation of rectal cancer. (n = 76).

Histogram Features ADC_aqp_ (μm^2^/ms)	Differentiation			*F*	*P*
	Good (n=13)	Moderate (n=53)	Poor (n=10)		
Mean	0.346 ± 0.030	0.388 ± 0.054	0.442 ± 0.090^**a,b**^	**8.046**	**0.001**
2.5^th^ Per	0.180 ± 0.078	0.173 ± 0.096	0.168 ± 0.054	0.053	0.949
25^th^ Per	0.293 ± 0.025	0.315 ± 0.051	0.338 ± 0.049	2.483	0.090
50^th^ per	0.337 ± 0.026	0.378 ± 0.054	0.421 ± 0.080^**a**^	**6.396**	**0.002**
75^th^ per	0.391 ± 0.035	0.451 ± 0.070^**a**^	0.537 ± 0.142^**a,b**^	**9.733**	**<0.001**
97.5^th^ per	0.545 ± 0.073	0.637 ± 0.115	0.777 ± 0.230^**a,b**^	**9.103**	**<0.001**
Kurtosis	1.422 ± 0.802	0.879 ± 0.921	0.927 ± 1.432	1.616	0.206
Skewness	0.420 ± 0.621	0.400 ± 0.452	0.436 ± 0.546	0.026	0.974

Per is the abbreviation of Percentile. LSD (Least Significant Difference) was used in a post hoc multiple comparisons of differentiation subgroups with histogram features of ADC_aqp_ value. Bonferroni correction was applied in multiple comparisons. Thus, *P* < 0.017 (0.05/3) was considered as a statistically significant difference. **a**: *P* < 0.017, vs good; **b**: *P* < 0.017, vs moderate.

**Figure 2:**
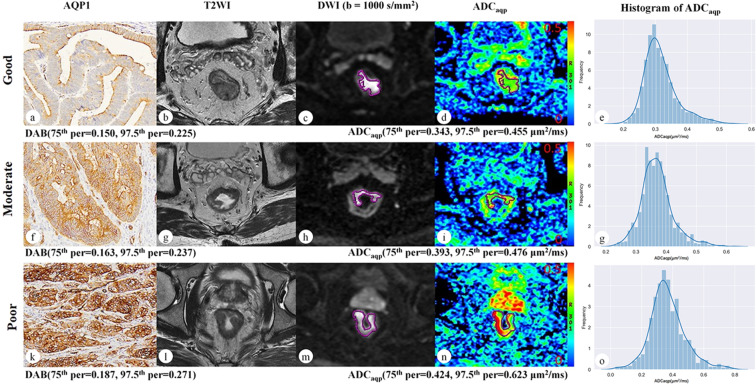
Representative digitized images of AQP1 IHC and corresponding MRI images of good (man, 65 y), moderate (man, 53 y) and poor (man, 57 y) differentiated rectal cancers. The staining intensity of AQP1 increased with the higher degree of tumor malignancy (**a**,**f**,**k**, ×100 magnification), similar to the ADC_aqp_ values (75^th^percentile and 97.5^th^percentile) (**d**,**i**,**n**). And the corresponding histograms of ADC_aqp_ values distribution within the whole tumor were generated for each rectal cancer (**e**,**g**,**o**).

As a typical example indicating the relationship of differentiation degrees of rectal cancer, AQP1 staining intensity and ADC_aqp_ value is shown in Fig. 2.

### ADC_aqp_, T stage and lymph node

Histogram features of ADC_aqp_ value showed no significant statistical differences in two subsets of T stage (T1–2 vs T3–4) and lymph node status (N+ vs N−) of rectal cancer (Table 3).

**Table 3:** The correlation between ADC_aqp_ histogram features and pathological features (T stage, lymph node status) of rectal cancer.

Histogram Features ADCaqp (μm^2^/ms)	T stage		*P*^*#*^	lymph Node		*P*^*#*^
	T1/2 (n = 22)	T3/4 (n = 54)		N− (n=39)	N+ (n = 37)	
Mean	0.385 ± 0.066	0.390 ± 0.060	0.779	0.390 ± 0.061	0.386 ± 0.062	0.790
2.5^th^ Per	0.155 ± 0.102	0.185 ± 0.085	0.193	0.170 ± 0.099	0.183 ± 0.081	0.533
25^th^ Per	0.308 ± 0.057	0.318 ± 0.048	0.436	0.316 ± 0.053	0.315 ± 0.048	0.890
50^th^ per	0.371 ± 0.063	0.379 ± 0.057	0.583	0.377 ± 0.062	0.376 ± 0.055	0.950
75^th^ per	0.453 ± 0.091	0.452 ± 0.084	0.978	0.455 ± 0.080	0.449 ± 0.091	0.784
97.5^th^ per	0.648 ± 0.161	0.636 ± 0.135	0.743	0.646 ± 0.124	0.632 ± 0.161	0.667
Kurtosis	1.036 ± 1.158	1.003 ± 1.070	0.907	1.080 ± 1.270	0.942 ± 0.870	0.586
Skewness	0.330 ± 0.555	0.465 ± 0.458	0.277	0.416 ± 0.563	0.436 ± 0.402	0.860

^*#*^Independent *t* test. N−: no metastatic lymph node, N+: metastatic lymph node.

## Discussion

Based on histogram analysis of both ultra-high b-value DWI and AQPs IHC, we found that there were positive correlations between AQP1 staining intensity and ADC_aqp_ value in three histogram features, which are mean, 75^th^ percentile and 97.5^th^ percentile (Fig. 1). However, neither AQP3 nor AQP5 were correlated with ADC_aqp_ value, which may indicate that AQP1 is mainly responsible for water transport through membranes in rectal cancer.

Previous studies found that colorectal cancer mainly expresses AQP1, 3 and 5^22^, but barely expresses AQP8^23^. Accordingly, the correlations between ADC_aqp_ and expressions of AQP1, 3 and 5 were investigated in this study. Tan *et al*.^16^ investigated the relationship between ultra-high b-value ADC and expressions of AQP1, 4 and 9 in cerebral astrocytoma, and found that ADC was only positively correlated with AQP4 expression. Wang *et al*.^17^ proved that positive correlation only existed between ultra-high b-value ADC and AQP2 expression, but not AQP1 or 4 in a rat model of diabetic nephropathy. These findings indicate that AQPs are diversely expressed at different organs and tumors in the role of water transport through membranes.

Water diffusivity and AQP1’s expression is widely and variously distributed due to the heterogeneity of tumors. It was found that the mean, 50^th^ percentile, 75^th^ percentile and 97.5^th^ percentile of ADC_aqp_ value significantly increased with a higher degree of malignance (Table 2). These results may illustrate that the more malignant part of a tumor harbors more water in terms of transmembrane movement with very slow speed, and this kind of abundant transmembrane movement of water needs more AQP expression or higher activity.

In this study, histogram analysis was applied in the scoring of IHC for the first time, and has been proved to be reliable because of a significant correlation with manual scoring (*r* = 0.685). The correlation coefficient of the 75^th^ percentile as well as the97.5^th^ percentile between ADC_aqp_ value and AQP1 staining intensity were greater than that of mean value. This finding advocates the fact that, compared to analysis of mean value, histogram analysis could estimate data distribution and tumor heterogeneity more reasonably and comprehensively^19^. And it has been confirmed that the AQP1 expression was significantly associated with numerous aggressive characteristics and differentiation was also associated with AQP1 expression in rectal cancer^24,25^, which means that tumors with more aggressive features will express more AQP1. Moreover, at high b-value rang of DWI the ADC value was more sensitive for the expression of AQP^26^, and high AQP1 expression could significantly increase the ADC value in several cell lines^5^. Therefore, we speculate that the lower percentile of ADC_aqp_ and AQP1’s expression (2.5^th^ and 25^th^) may represent the tumor matrix which is lacking in tumor cells and low AQP1 expression, or the part of good differentiated tumor cells which presents indistinguishable ADC_aqp_ and AQP1 expression in different patients. Meanwhile, the higher percentile of ADC_aqp_ and AQP1’s expression (75^th^ and 97.5^th^) may represent the most malignant part of a tumor, which features remarkable differences among different patients.

As the AQP1 is associated with the response to adjuvant chemotherapy in stage II and III colorectal cancer^24^, while AQP1 channel blocker could suppress the invasiveness of colon cells^27^. These results implied that AQP1 could be a biomarker for predicting and monitoring the treatment response or a therapeutic target. An imaging parameter which can reflect the AQP1 expression will be a useful marker in clinical application, especially in response prediction and tumor surveillance. In the current results, only 75th percentile and 97.5th percentile exhibited significant correlation with AQP1 expression (*r* = 0.323 and 0.362, respectively, both *P* < 0.05, Fig. 1). And we speculate that higher percentile of ADC_aqp_ may present more malignant cells which features remarkable differences among different patients and is the decisional factor for prognosis of cancer. Therefore, considering the remarkable value of AQP1 in clinical application, we recommend these two parameters as the markers in future research.

The result of pairwise comparison showed the 75^th^ percentile of ADC_aqp_ had greater discriminatory power than other histogram features of ADC_aqp_ in distinguishing the good, moderate and poor differentiated subgroups. However, according to the current results, histogram features of ADC_aqp_ value showed no significant differences in two subsets of T stage and lymph node status of rectal cancer. This indicated that the ADC_aqp_ value was only correlated with the grade of tumor differentiation, but not with other prognostic indicators of rectal cancer according to the existing data. As for kurtosis, the poor ICCs of this parameter of the AQP1 score may be one of the factors accounting for no relationship with ADC_aqp_ value.

This research has several limitations apart from small sample size, especially good and poor differentiated subjects. Firstly, selected b-values and effective diffusion time of ultra-high b-value DWI could impact significantly on detecting the movement of water transmembrane transport. These two crucial factors should be explored in further studies. Secondly, water transmembrane movement is not only mediated by AQP1, but also by AQP3 and 5, or other AQPs which have not been confirmed. This research investigated the correlation between ADC_aqp_ and AQP1, 3 and 5 independently, but not comprehensively. Therefore, a more complex mathematical model and elaborately designed research should be developed to address these problems. Thirdly, although the histogram analysis applied in the scoring of AQP staining intensity has been proven to be reliable, there are still some drawbacks (time consumption, inevitable residual of nonspecific staining area which should have been erased).A more intelligent and automatic process module in evaluating the intensity of AQP staining should be developed. In addition, the histogram features of ADC_aqp_ were generated from a whole tumor, while the histogram features of AQP expression were acquired from a single slice. Therefore, it is difficult to analyze the correlation between ADC_aqp_ and AQP expression by point to point manner. At certain extent, the careful selection of tumor sample by our experienced pathologist could offset the negative impact from the comparison the whole tumor on imaging with single slice IHC.

In conclusion, the mean, 75^th^ percentile and 97.5^th^ percentile of ADC_aqp_ value derived from ultra-high b-value DWI could reflect AQP1’s expression and the malignancy degree of rectal cancer based on histogram analysis, and the 75^th^ percentile and 97.5^th^ percentile performed better. Ultra-high b-value DWI may provide an alternative form of non-invasive imaging marker of AQP1 expression in rectal cancer.

## Methods

### Patient selection

**Figure 3:**
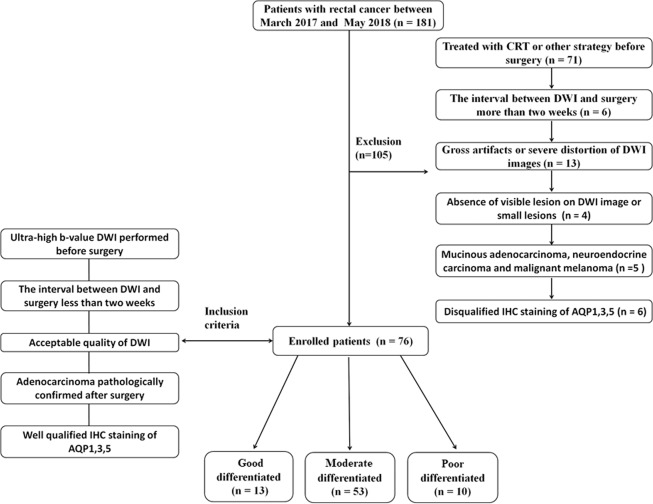
Flowchart showing the patient selection process.

This prospective study was approved by the Ethical Review Board of Xijing hospital and conducted in accordance with the Declaration of Helsinki, and written consents were obtained from all participants. From March 2017 to May 2018, 181 rectal cancer patients confirmed by colonoscopy biopsy were enrolled and underwent ultra-high b-value DWI. Altogether 76 subjects, 48 men (age, 62.3 ± 10.78) and 28 women (age, 61.0 ± 11.6), were finally selected according to the following inclusion criteria: *(a)*ultra-high b-value DWI performed before surgery; *(b)* the interval between DWI and surgery was less than two weeks; *(c)* acceptable quality of diffusion-weighted images; *(d)* adenocarcinoma pathologically confirmed after surgery; *(e)* well qualified immunohistochemistry (IHC) staining of paraffin embedded samples of AQP1, 3 and 5. The other 105 patients were excluded based on the following exclusive criteria: *(a)* treated with radio-chemotherapy (CRT) or other strategies before surgery (n = 71); *(b)* the interval between DWI and surgery was more than two weeks (n = 6); *(c)* gross artifacts or severe distortion of DWI images (n = 13); *(d)* absence of visible lesion on diffusion-weighted image or the area of lesion’s ROI was less than 100mm^2^ on the largest axial plane (n = 4); *(e)* other pathological types of tumor (mucinous adenocarcinoma, neuroendocrine carcinoma and malignant melanoma) (n = 5); *(f)*disqualified IHC staining of AQP1,3 and 5 (n = 6). Enrolled rectal cancer subjects were divided into 3 groups based on the degree of differentiation by one experienced gastrointestinal pathologist (10 years of experience) according to the WHO classification system^28^: good (n = 13), moderate (n = 53) and poor (n = 10). Other pathological indicators [T stage (T1–2 vs T3–4) and lymph node status (N+ vs N−)] were assessed according to the TNM staging system of the 7^th^ edition of the American Joint Committee on Cancer^29^. The patient selection process is shown in Fig. 3.

### MRI acquisition

All scans were performed on a 3.0 T MR scanner (Discovery MR750, GE Medical Systems) with an 8-channel phased-array coil. Bowel preparation was conducted by drinking folium sennae soup (a kind of laxative) after dinner on the night before the examination. Antispasmodic and other intestinal contrast agents were not used. Rectal MRI protocols included axial T1WI (TR/TE = 487/8), coronal and sagittal T2WI (TR/TE = 7355/136), axial FRFSE T2WI (TR/TE = 7096/133) with small FOV (220 mm), routine DWI (b = 0, 1000 s/mm^2^), and ultra-high b-value DWI (single-shot SE-EPI diffusion-weighted sequence) with 5 b-values of 1700, 2000, 2500, 3000 and 3500 s/mm^2^ (TR/TE = 4431/71 ms, FOV = 260×260 mm^2^, matrix = 128×128, slice thickness = 5 mm, intersection gap = 0.5 mm, NEX = 4 to 8 (increasing with b-values), total scan time of ultra-high b-value DWI was about 7 minutes). Axial images both for routine DWI and ultra-high b-value DWI were designed to cover the entire lesion area with the same spatial prescription.

### Ultra-high b-value DWI analysis

ADC_aqp_ images were generated with the pixel-wise mono-exponential interpolation^4^ of five ultra-high b-value DWI images(b-values =1700, 2000, 2500, 3000 and 3500 s/mm^2^) according to Eq. (1) by using the AQP module build-in Functional Tool of GE Advanced Workstation 4.6.

1 $${\rm{S}}({\rm{b}})/{{\rm{S}}}_{0}=\exp (-{\rm{b}}\bullet {{\rm{ADC}}}_{{\rm{aqp}}}),\,{\rm{b}}\ge 1700\,{\rm{s}}/{{\rm{mm}}}^{2}$$

Because of the high CNR^30^ (contrast noise ratio) of tumor to normal rectal wall in DWI with b = 1000 s/mm^2^,the ROI of a whole tumor was delineated on each layer of the DW images (b = 1000 s/mm^2^) by two abdominal MRI radiologists (one with 6 years of experience; the other with 5 years of experience) using IKT-SNAP (version 3.6.0)^31^. Meanwhile the artifact signal parts were excluded. The ROI of the whole tumor was saved as a NIfTI-1 data format file as a mask. The histogram features of the whole tumor were extracted by importing the ADC_aqp_ image and the corresponding mask into third-party software (AK version 3.0.1, GE Healthcare). As a result, eight histogram features for the tumor were generated: mean, 2.5^th^ percentile, 25^th^ percentile, 50^th^ percentile, 75^th^ percentile, 97.5^th^ percentile, kurtosis and skewness of ADC_aqp_ value.

### AQP immunohistochemistry

All cancer samples were carefully selected by two experienced pathologists (one with 8 years of experience, another with 10 years of experience) before IHC staining. IHC staining was performed by using a Leica BOND-MAX auto-stainer (Leica Instrument Co., Ltd.), and the AQP1 antibody (ab168387, Abcam) was diluted to 1: 1000. IHC was conducted as follows: firstly, 4-micron thick tissue sections were cut in microtome, deparaffinized in xylene, rehydrated through graded ethanol (100% and 95%), and rinsed in water. Then the sections were subjected to heat-induced antigen retrieval, and finally loaded onto the Benchmark auto-stainer. Detection was performed using a bond polymer refine detection kit (Leica Instrument Co., Ltd.). The process of IHC of AQP3 (ab168387, Abcam, 1:200) and AQP5 (ab168387, Abcam, 1:500) was similar to the description above.

### Scoring of AQP immunohistochemistry

Two IHC scoring methodologies were used in this study. The first is that the protein expressions of AQP1, 3 and 5 were quantified as a percentage (range 0-100%) of positive cells presented among all tumor cells. The positive cell ratio score is based on the following criteria: 0 (0%), 1 (1–25%), 2 (26–50%), 3 (51–75%), 4 (>75%).The staining intensity of AQP1,3 and 5 was evaluated with reference to the normal mucosal epithelium and scored as 0, 1+, 2+ and 3+. The final statistical measure is the staining intensity score + positive cell ratio score and is termed as the manual AQP score^14^. Each of the sections was scored by two observers, who were unaware of the patient’s medical information, and the mean value of scores from the two observers for each section was submitted for statistical analysis.

The second method is that the protein expression of AQPs was estimated by QuPath (open source software for Quantitative Pathology, version 0.1.2)^32^.Digitized pathological images of each IHC slice of AQPs were acquired at ×100 magnification with an Olympus slide scanner (Olympus motorized BX61VS). The ROI of the tumor was delineated on the pathological image with nonspecific staining erased, and the ROI was tiled into thousands of tiles after staining background correction. Each tile was 100 μm × 100 μm in size. The mean of the DAB (3, 3’-Diaminobenzidine) intensity of each tile within the ROI was calculated with QuPath and analyzed by SPSS for histogram features calculation (mean, 2.5^th^ percentile, 25^th^ percentile, 50^th^ percentile, 75^th^ percentile, 97.5^th^ percentile, kurtosis and skewness). The process of histogram analysis of AQP staining is shown in Supplemental Material 1.

### Statistical analysis

First, inter-observer reproducibility was tested for all quantitative parameters with ICC (interclass correlation coefficient). The distribution of quantitative data was tested by using the Kolmogorov-Smirnov test. The normally distributed data were expressed as mean ± standard deviation. One-way ANOVA (analysis of variance) was employed to evaluate the histogram features of ADC_aqp_ value among three differentiation groups (good, moderate and poor). Pearson’s correlation analysis was conducted to test the relationship between histogram features of ADC_aqp_ and scoring of AQP IHC both by QuPath and manually. The differences of histogram features of ADC_aqp_ images in two groups of other pathological indicators [T stage (T1–2 vs T3–4) and lymph node status (N+ vs N−)] were analyzed by independent *t* test. All statistical analyses were performed by SPSS (version 19.0; Inc.). A *P* value of less than 0.05 was considered statistically significant.

## Supplementary information

Supplementary Information.

Supplementary Information2.

Supplementary Information3.
