# Supplementary material for: Based on Histogram Analysis: ADCaqp Derived from Ultra-high b-Value DWI could be a Non-invasive Specific Biomarker for Rectal Cancer Prognosis
Source: Sci Rep. 2020 Jun 23;10:10158. doi: 10.1038/s41598-020-67263-4 (PMC7311405; doi:10.1038/s41598-020-67263-4)
Supplement: Supplementary file 1 — Supplementary Information. [file 41598_2020_67263_MOESM1_ESM.pdf]

Supplementary Table S1. Correlation between ADC_{aqp} and staining intensity of AQP3, AQP5 based on histogram features analysis with QuPath (n=76).

Histogram Features	ADC_{aqp} ($\mu m^2/ms$)	AQP3			AQP5		
		SI	***r1***	***P1***	SI	***r2***	***P2***
Mean	0.388±0.062	0.090±0.040	-0.053	0.648	0.124±0.078	-0.003	0.977
2.5^{th} Per	0.174±0.088	0.066±0.010	-0.036	0.755	0.069±0.008	0.039	0.739
25th Per	0.315±0.049	0.077±0.018	-0.065	0.575	0.085±0.028	-0.009	0.936
50th per	0.376±0.059	0.086±0.034	-0.048	0.678	0.108±0.072	0.042	0.721
75th per	0.452±0.087	0.099±0.058	-0.050	0.666	0.144±0.119	0.024	0.834
97.5^{th} per	0.640±0.143	0.138±0.110	-0.121	0.299	0.270±0.219	-0.030	0.799
Kurtosis	0.978±0.989	34.651±69.604	-0.079	0.500	20.421±35.807	0.106	0.364
Skewness	0.408±0.489	2.771±2.337	-0.133	0.259	2.736±1.956	0.043	0.709

Per is the abbreviation of Percentile.SI: staining intensity; *r1:*Pearson correlation coefficient between ADC_{aqp} and staining intensity of AQP3; *r2:*Pearson correlation coefficient between ADC_{aqp} and staining intensity of AQP5.
