# Supplementary material for: Based on Histogram Analysis: ADCaqp Derived from Ultra-high b-Value DWI could be a Non-invasive Specific Biomarker for Rectal Cancer Prognosis
Source: Sci Rep. 2020 Jun 23;10:10158. doi: 10.1038/s41598-020-67263-4 (PMC7311405; doi:10.1038/s41598-020-67263-4)

Supplemental Material 1.Flow chart of AQP staining estimation with QuPath (version 0.1.2)

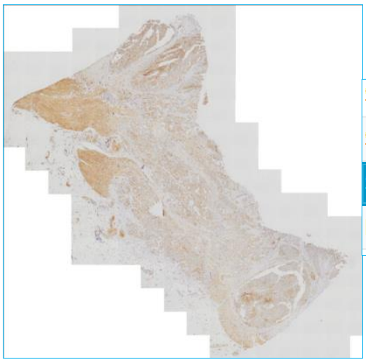

Stain 1	Hematoxylin: 0.651 0.701 0.29
Stain 2	DAB: 0.269 0.568 0.778
Stain 3	Residual: 0.633 -0.713 0.302
Background	255 255 255

STEP1: Correct the staining background with “Estimate stain vectors” tool

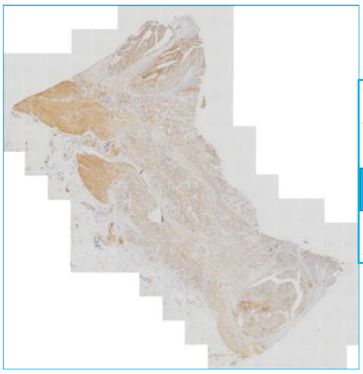

Stain 1	Hematoxylin: 0.546 0.793 0.2...
Stain 2	DAB: 0.233 0.447 0.864
Stain 3	Residual: 0.807 -0.584 0.085
Background	212 212 212

STEP2: Draw ROI of tumor and any part of nonspecific staining erased

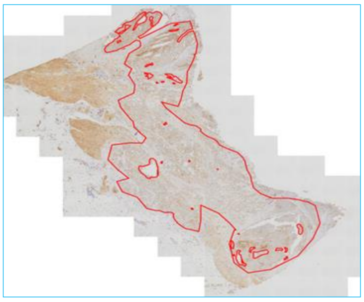

STEP3: Create tiles within ROI

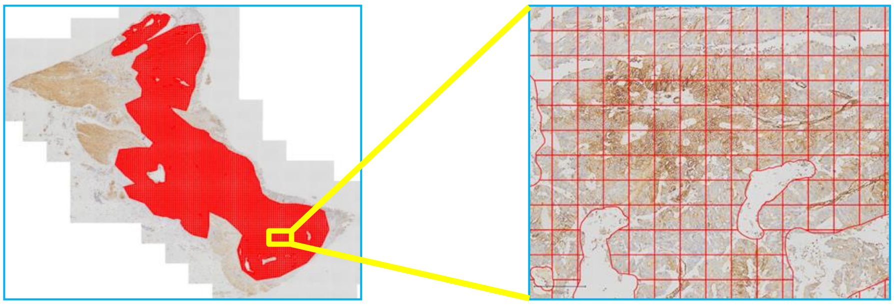

STEP4: Calculate mean DAB intensity of each tile

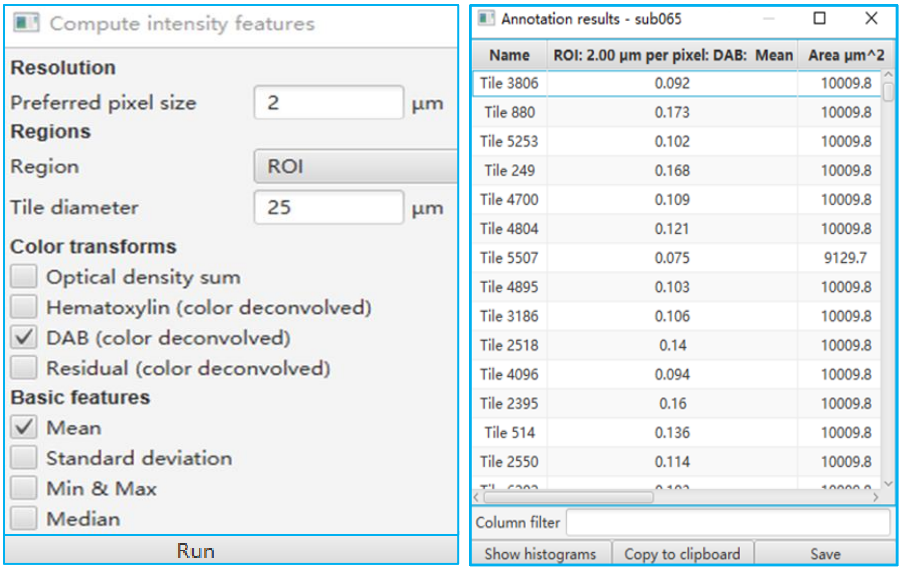

STEP5: Calculate histogram features of DAB intensity

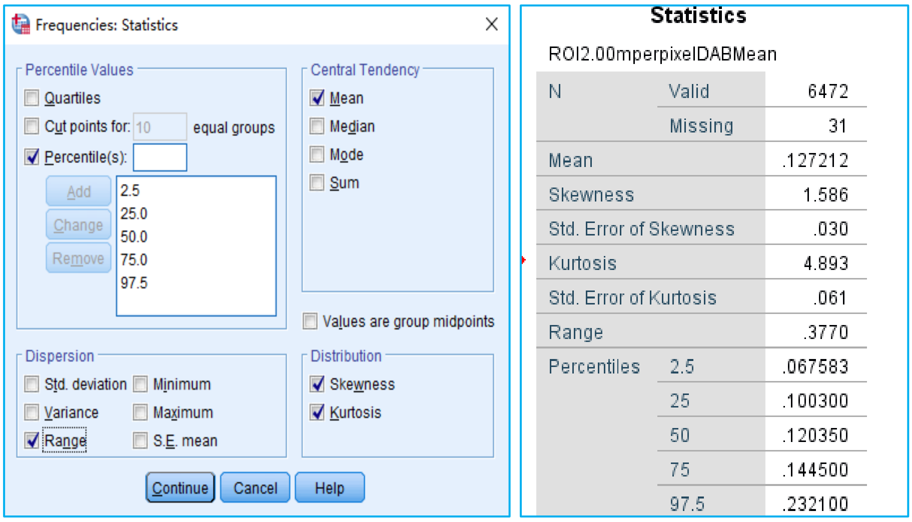

Supplement: Supplementary file 2 — Supplementary Information2. [file 41598_2020_67263_MOESM2_ESM.pdf]
