# Supplementary material for: Based on Histogram Analysis: ADCaqp Derived from Ultra-high b-Value DWI could be a Non-invasive Specific Biomarker for Rectal Cancer Prognosis
Source: Sci Rep. 2020 Jun 23;10:10158. doi: 10.1038/s41598-020-67263-4 (PMC7311405; doi:10.1038/s41598-020-67263-4)
Supplement: Supplementary file 3 — Supplementary Information3. [file 41598_2020_67263_MOESM3_ESM.pdf]

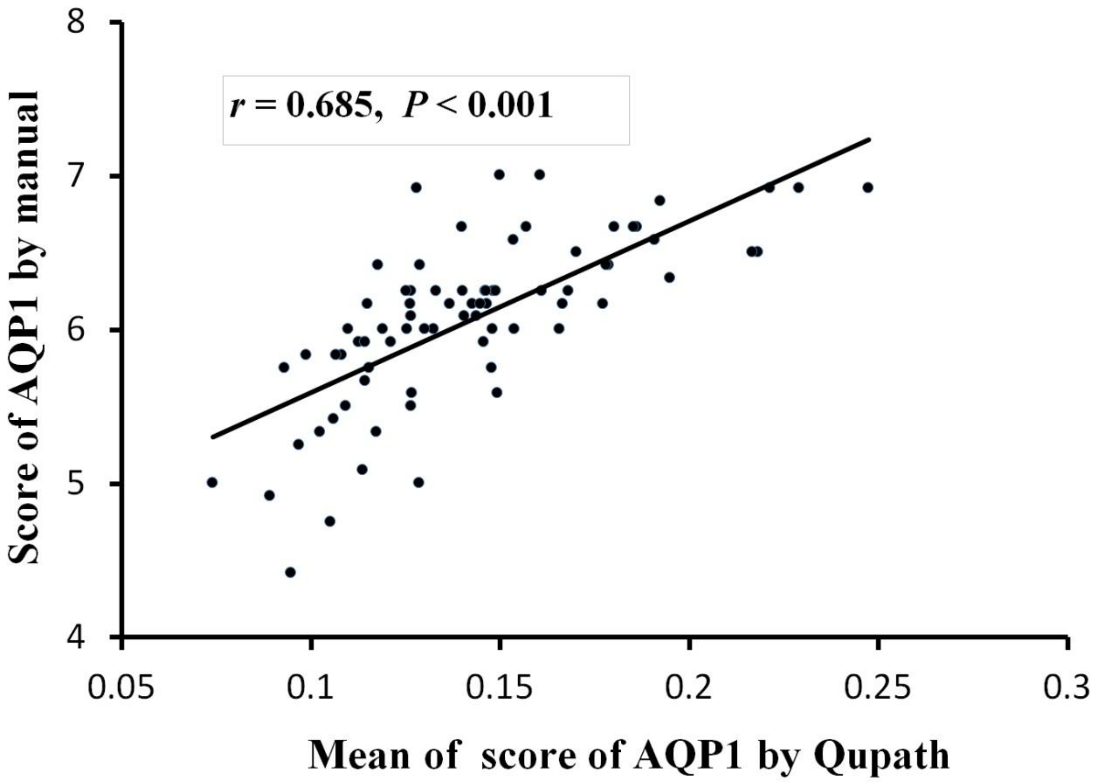

Supplementary Figure S1. Correlation of the mean AQP1 staining score between QuPath and manual method.
